# Identification of benzopyrone as a common structural feature in compounds with anti-inflammatory activity in a zebrafish phenotypic screen

**DOI:** 10.1242/dmm.024935

**Published:** 2016-06-01

**Authors:** Anne L. Robertson, Nikolay V. Ogryzko, Katherine M. Henry, Catherine A. Loynes, Matthew J. Foulkes, Marco M. Meloni, Xingang Wang, Christopher Ford, Malcolm Jackson, Philip W. Ingham, Heather L. Wilson, Stuart N. Farrow, Roberto Solari, Roderick J. Flower, Simon Jones, Moira K. B. Whyte, Stephen A. Renshaw

**Affiliations:** 1 The Bateson Centre, University of Sheffield, Sheffield, S10 2TN, UK; 2 Department of Infection, Immunity and Cardiovascular Disease, University of Sheffield, Sheffield, S10 2TN, UK; 3 Stem Cell Program and Division of Hematology/Oncology, Children's Hospital Boston, Howard Hughes Medical Institute, Harvard Stem Cell Institute, Harvard Medical School, Boston, 02115 MA, USA; 4 Department of Chemistry, University of Sheffield, Sheffield, S10 2TN, UK; 5 Wishtech Medical Technology, Weihai, Shandong, 264200, China; 6 Department of Musculoskeletal Biology, Institute of Ageing and Chronic Disease, University of Liverpool, Liverpool, L7 9TX, UK; 7 MRC Arthritis Research UK Centre for Integrated Research into Musculoskeletal Ageing, University of Liverpool, Liverpool, L7 9TX, UK; 8 Institute of Molecular and Cell Biology, 61 Biopolis Drive, Proteos, Singapore, 138673, Singapore; 9 Institute of Human Development, University of Manchester, Manchester, M13 9PL, UK; 10 Faculty of Medicine, National Heart and Lung Institute, Imperial College London, Norfolk Place, London, W2 1NY, UK; 11 William Harvey Research Institute, Queen Mary University of London, Charterhouse Square, London, EC1M 6BQ, UK; 12 MRC/UoE Centre for Inflammation Research, University of Edinburgh, The Queen's Medical Research Institute, Edinburgh, EH16 4TJ, UK

**Keywords:** Zebrafish, Inflammation, Neutrophil apoptosis, Chromone, Benzopyrone

## Abstract

Neutrophils are essential for host defence and are recruited to sites of inflammation in response to tissue injury or infection. For inflammation to resolve, these cells must be cleared efficiently and in a controlled manner, either by apoptosis or reverse migration. If the inflammatory response is not well-regulated, persistent neutrophils can cause damage to host tissues and contribute to the pathogenesis of chronic inflammatory diseases, which respond poorly to current treatments. It is therefore important to develop drug discovery strategies that can identify new therapeutics specifically targeting neutrophils, either by promoting their clearance or by preventing their recruitment. Our recent *in vivo* chemical genetic screen for accelerators of inflammation resolution identified a subset of compounds sharing a common chemical signature, the bicyclic benzopyrone rings. Here, we further investigate the mechanisms of action of the most active of this chemical series, isopimpinellin, in our zebrafish model of neutrophilic inflammation. We found that this compound targets both the recruitment and resolution phases of the inflammatory response. Neutrophil migration towards a site of injury is reduced by isopimpinellin and this occurs as a result of PI3K inhibition. We also show that isopimpinellin induces neutrophil apoptosis to drive inflammation resolution *in vivo* using a new zebrafish reporter line detecting *in vivo* neutrophil caspase-3 activity and allowing quantification of flux through the apoptotic pathway in real time. Finally, our studies reveal that clinically available ‘cromones’ are structurally related to isopimpinellin and have previously undescribed pro-resolution activity *in vivo*. These findings could have implications for the therapeutic use of benzopyrones in inflammatory disease.

## Abstract (precis)

**Summary:** Zebrafish inflammation screen identifies a new series of structurally related compounds with combined anti-inflammatory and pro-resolution activity, and reveals a previously unknown mechanism of action of clinical cromones.

## INTRODUCTION

The acute inflammatory response is an essential host defence mechanism and is initiated by the innate immune system in the event of tissue injury or infection. It is a highly controlled sequence of events that involves the coordinated activity of multiple cytokines, lipid mediators and cell types, and can be broadly split into three phases: recruitment, peak inflammation and resolution (Serhan et al., 2007). One of the most important innate immune cells involved in the inflammatory response is the neutrophil. During the recruitment phase, these cells are activated in response to inflammatory stimuli and migrate to inflamed tissue, following gradients of soluble chemokines such as interleukin-8 (IL-8 or CXCL8) (Medzhitov, 2008). This chemokine signals via CXCR2 receptors on the neutrophil surface to activate downstream pathways, including the phosphatidylinositol 3-kinase (PI3K) pathway that is required for neutrophil chemotaxis (Ferguson et al., 2007; Hirsch et al., 2000; Yoo et al., 2010). Once at the site of inflammation, neutrophils eliminate any invading pathogens by phagocytosis, degranulation and the production of reactive oxygen species and extracellular traps (Fox et al., 2010). Inflammation must then resolve in order to restore homeostasis and promote tissue repair. During the resolution phase, neutrophils are removed either by undergoing apoptosis and engulfment by macrophages, or by leaving the site of inflammation by reverse migration (Henry et al., 2013).

If inflammation is not well-regulated, persistent neutrophilic inflammation can cause host tissue damage and chronic inflammation, which can contribute to the pathogenesis of diseases such as chronic obstructive pulmonary disease (COPD), rheumatoid arthritis and atherosclerosis (Serhan et al., 2007). Many of these are characterised by unresolved neutrophilic inflammation and respond poorly to current therapies, making the neutrophil a key target for drug discovery approaches. Potentially, drugs used to treat inflammatory disease might act either by preventing the further recruitment and accumulation of neutrophils at inflammatory sites (‘anti-inflammatory’) or by promoting neutrophil clearance to drive inflammation resolution (‘pro-resolution’).

The zebrafish (*Danio rerio*) is a powerful model for the study of vertebrate biology. The development of transgenic lines labelling innate immune cells has enabled *in vivo* investigation of the mechanisms regulating the different phases of the inflammatory response (Ellett et al., 2011; Hall et al., 2007; Mathias et al., 2006; Renshaw et al., 2006). With its transparent larvae, small size and high fecundity, the zebrafish model lends itself particularly well to drug discovery by high-throughput chemical genetic screening, and multiple success stories are emerging using this unique whole-organism approach (Hall et al., 2014; North et al., 2007; Takaki et al., 2012; Tamplin et al., 2015; Wang et al., 2014).

We recently described a chemical genetic screen for accelerators of inflammation resolution, in which we found the first compound that could promote neutrophil reverse migration to drive inflammation resolution *in vivo* (Robertson et al., 2014). Here, we investigate the mechanism of action of the largest described, structurally distinct subset of active compounds from an *in vivo* chemical genetic anti-inflammatory screen. These compounds share both structural and functional similarity and their activity is two-fold: neutrophil recruitment is inhibited and inflammation resolution is accelerated. We also describe a new zebrafish reporter line for *in vivo* neutrophil caspase-3 activity, which allows us to visualise neutrophil apoptosis during inflammation resolution in real time. Finally, our studies led to the discovery of a previously undescribed mechanism of action for a group of clinically available therapeutics, the cromones, which could impact on their use in inflammatory disease.

## RESULTS

### Zebrafish inflammation drug screen identifies a chemical series with structural and functional similarity

**Fig. 1.**
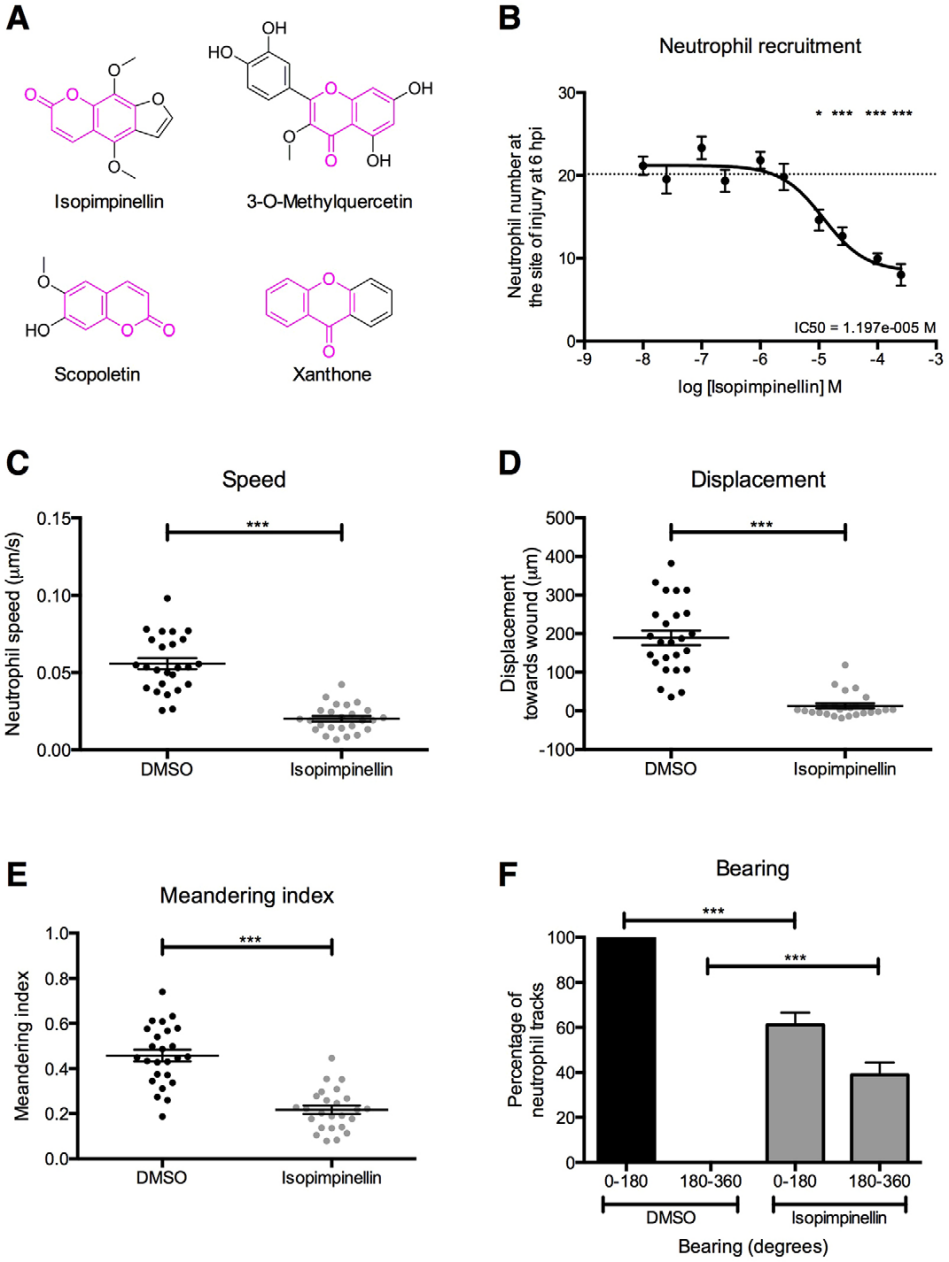
**Isopimpinellin inhibits neutrophil migration towards tissue injury.** (A) Isopimpinellin shares structural similarity to three other compounds identified in our previous drug screen for accelerators of inflammation resolution (Robertson et al., 2014). Common benzopyrone structures are highlighted in magenta. (B) Recruitment assay in *mpx:GFP* larvae treated with varying doses of isopimpinellin immediately post-injury. Isopimpinellin significantly reduces neutrophil numbers at the wound at 6 hpi in a dose-dependent manner (one-way ANOVA with Dunnett's multiple-comparison post-test; *P<0.05, ***P<0.001; *n*=18, performed as three independent experiments). Dotted line at *y*=20.17 indicates mean neutrophil number at wound in DMSO control larvae. (C-F) Neutrophil tracking assay in *mpx:GFP* larvae pretreated with DMSO or 25 μM isopimpinellin for 2 h prior to tail fin-injury and time-lapsed from 1 to 3 hpi. Individual neutrophils were tracked manually to analyse (C) speed, (D) displacement towards the wound and (E) meandering index. All were reduced in isopimpinellin-treated larvae compared to DMSO controls (unpaired *t*-test; ***P<0.001; *n*=26, performed as three independent experiments). Data points represent mean of six tracked neutrophils per fish. For bearing (F), angles of 0° to 180° indicate migration towards the wound, whereas 180° to 360° indicate migration away from the wound (one-way ANOVA with Bonferroni's multiple-comparison post-test to compare selected columns; ***P<0.001; *n*=30, performed as three independent experiments).

We recently established a drug screening assay in a transgenic zebrafish model of acute inflammation and identified 21 new pro-resolution compounds (Robertson et al., 2014). Structural similarity comparisons revealed that a particular chemical group, consisting of fused benzene and pyran rings with an attached carbonyl group, was common to a subset of these. Commonly referred to as ‘chromone’ (1,4-benzopyrone), this group or its isomer ‘coumarin’ (1-benzopyran-2-one) is present in four of the nine most-active pro-resolution compounds identified in our screen (Fig. 1A). All four of these significantly accelerated inflammation resolution in our zebrafish tail-fin injury model and three of them also inhibited neutrophil recruitment (Robertson et al., 2014). To further investigate the functionality of benzopyrone derivatives, we tested a further ten commercially available related compounds and also synthesised a series of five analogue compounds for testing in our zebrafish inflammation resolution assay. Larvae were treated with compounds once inflammation was already established at 6 hours post-injury (hpi) and their effects on neutrophil number were assessed at 12 hpi. All of the commercially available compounds significantly reduced neutrophil numbers at the wound (Fig. S1), along with three of our newly synthesised analogues (Fig. S2). To explore the potential significance of this functional group in accelerating inflammation resolution, we selected the most active of the benzopyrone subset identified in our screen, isopimpinellin, for mechanistic investigation.

### Isopimpinellin inhibits neutrophil recruitment *in vivo*

Isopimpinellin is a naturally occurring coumarin found in plants of the Apiaceae family. It has been studied for its potential anti-carcinogenic properties (Kleiner et al., 2002; Prince et al., 2006), but there is currently no published evidence to explain its anti-inflammatory activity. We initially investigated the effect of isopimpinellin on the recruitment phase of the inflammatory response in the tail-fin injury assay, by treating zebrafish larvae immediately after wounding. At 6 hpi, we found that neutrophil number at the wound was reduced in isopimpinellin-treated larvae compared to controls, in a concentration-dependent manner (Fig. 1B). When individual neutrophils were tracked during the recruitment phase of inflammation, we detected a decrease in neutrophil speed (Fig. 1C), displacement (the linear distance each neutrophil travelled towards the wound) (Fig. 1D) and meandering index (the displacement divided by the total length of the neutrophil track) (Fig. 1E). We also found a difference in bearing (the angle of movement towards the wound) between the two groups, with fewer neutrophils moving towards the wound in the presence of isopimpinellin (Fig. 1F). These data suggest that isopimpinellin alters the migratory behaviour of neutrophils, such that they move more slowly and with less directionality, resulting in reduced recruitment towards the chemokine gradient at the wound.

### Isopimpinellin inhibits neutrophil recruitment upstream of phosphatidylinositol 3-kinases

**Fig. 2.**
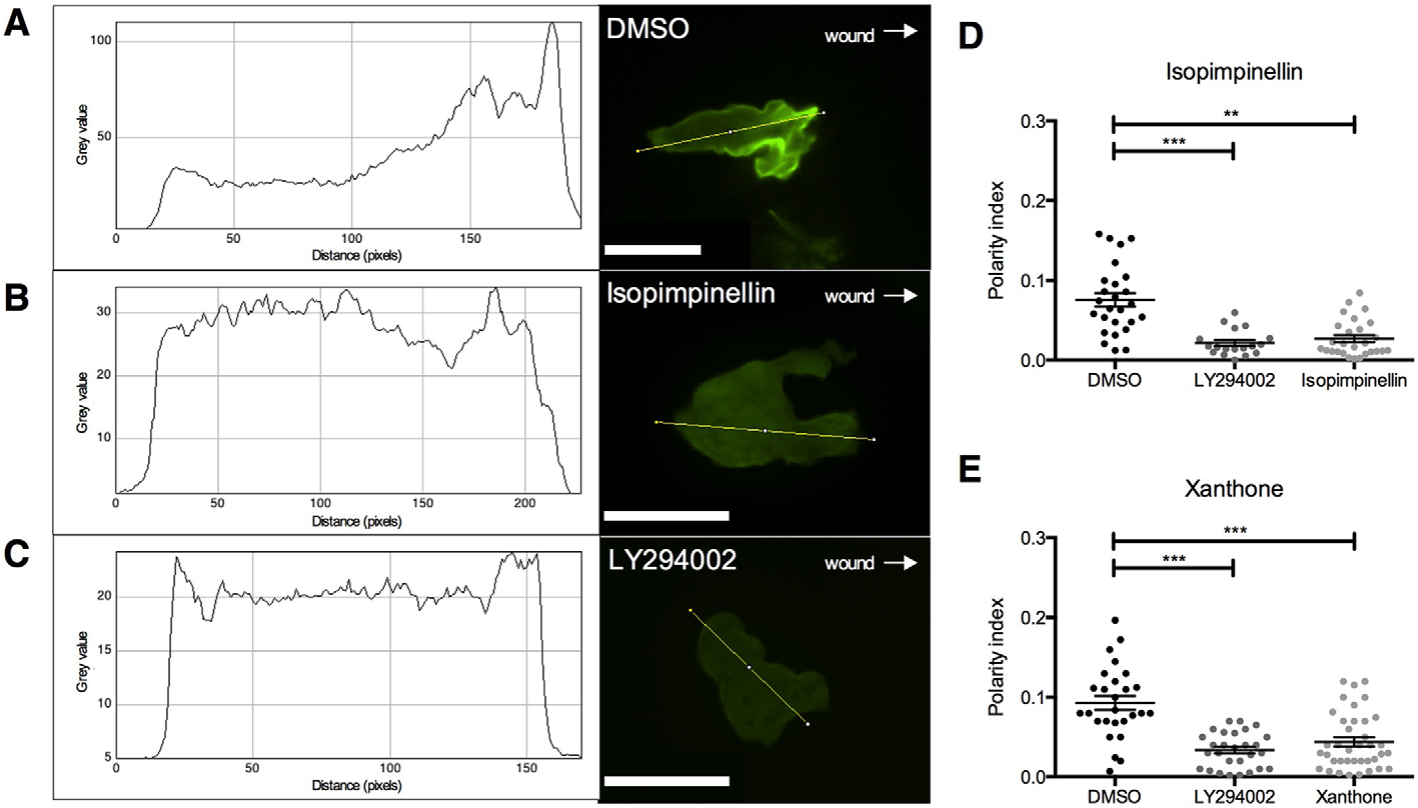
**Isopimpinellin and xanthone inhibit polarisation of neutrophils upstream of PI3K.** Assay to measure the polarity index of neutrophils in *Tg(lyz:PHAkt-EGFP)* larvae pretreated with 25 μM isopimpinellin, 25 μM xanthone, 50 μM LY294002 or DMSO as a vehicle control for 2 h prior to tail-fin injury. Representative images of individual neutrophils in the region between the wound site and posterior blood island illustrate polarisation and a defined leading edge of a migrating neutrophil in (A) DMSO control-treated larvae, compared to (B) isopimpinellin- and (C) LY294002-treated larvae, which do not polarise or have a defined leading edge (scale bars: 11 μm). Arrows indicate direction of wound. Fluorescence (referred to as grey value) was measured in a transection of each neutrophil (the yellow lines drawn through the cells) to generate intensity profiles shown in the panels on the left and quantify neutrophil polarity index as previously described (Wang et al., 2014). Both (D) isopimpinellin and (E) xanthone reduced the neutrophil polarity index to a similar level as the known PI3K inhibitor LY294002 (one-way ANOVA with Dunnett's multiple-comparison post-test; **P<0.01, ***P<0.001; *n*=27, performed as three independent experiments).

In our previous study, we compared the activity of the positive hits identified in our screen with a panel of compounds with known effects on inflammatory signalling pathways, such as inhibitors of PI3K and mitogen-activated protein kinase (MAPK), using hierarchical cluster analysis (Robertson et al., 2014). This indicated that the activity of isopimpinellin in our zebrafish inflammation assays correlated with a pan-inhibitor of PI3K, ZSTK474. *In vivo* evidence suggests that PI3K regulates neutrophil polarity and that its activation at the leading edge of cells is required for actin polymerisation during chemotaxis (Yoo et al., 2010). This is dependent on the tightly controlled spatial and temporal accumulation of phosphatidylinositol (3,4,5)-triphosphate (PIP_3_), protein kinase B (Akt) and actin at the leading edge, a process that is impaired in neutrophils lacking PI3Kγ (Hannigan et al., 2002; Ferguson et al., 2007). Based on the correlation between isopimpinellin and ZSTK474 revealed by cluster analysis, we predicted that the effect of our hit compound on neutrophil migration during the recruitment phase of inflammation was a result of PI3K inhibition and loss of the intracellular polarity required to direct migration. To investigate this, we used a fluorescent reporter line, which labels the pleckstrin homology (PH) domain of Akt with EGFP to permit visualisation of localised PI3K activity *in vivo* (Burgon et al., 2014; Wang et al., 2014). In vehicle control-treated larvae, the EGFP signal accumulated at the leading edge of neutrophils as they migrated towards the wound shortly after tail-fin injury (Fig. 2A). In contrast, neutrophils from isopimpinellin-treated larvae did not migrate as readily to the wound and most of these did not have a defined leading edge (Fig. 2B), displaying a morphology characteristic of neutrophils from larvae exposed to the PI3K inhibitor LY294002 (Fig. 2C). Using a numerical measure of cell polarity (‘polarity index’) (Wang et al., 2014), we found that isopimpinellin reduced neutrophil polarity to a level comparable to LY294002 (Fig. 2D). We also examined the effect of another compound from our benzopyrone subset, xanthone, finding a similar reduction in neutrophil polarity (Fig. 2E).

### Isopimpinellin induces apoptosis of neutrophils during inflammation resolution *in vivo*

**Fig. 3.**
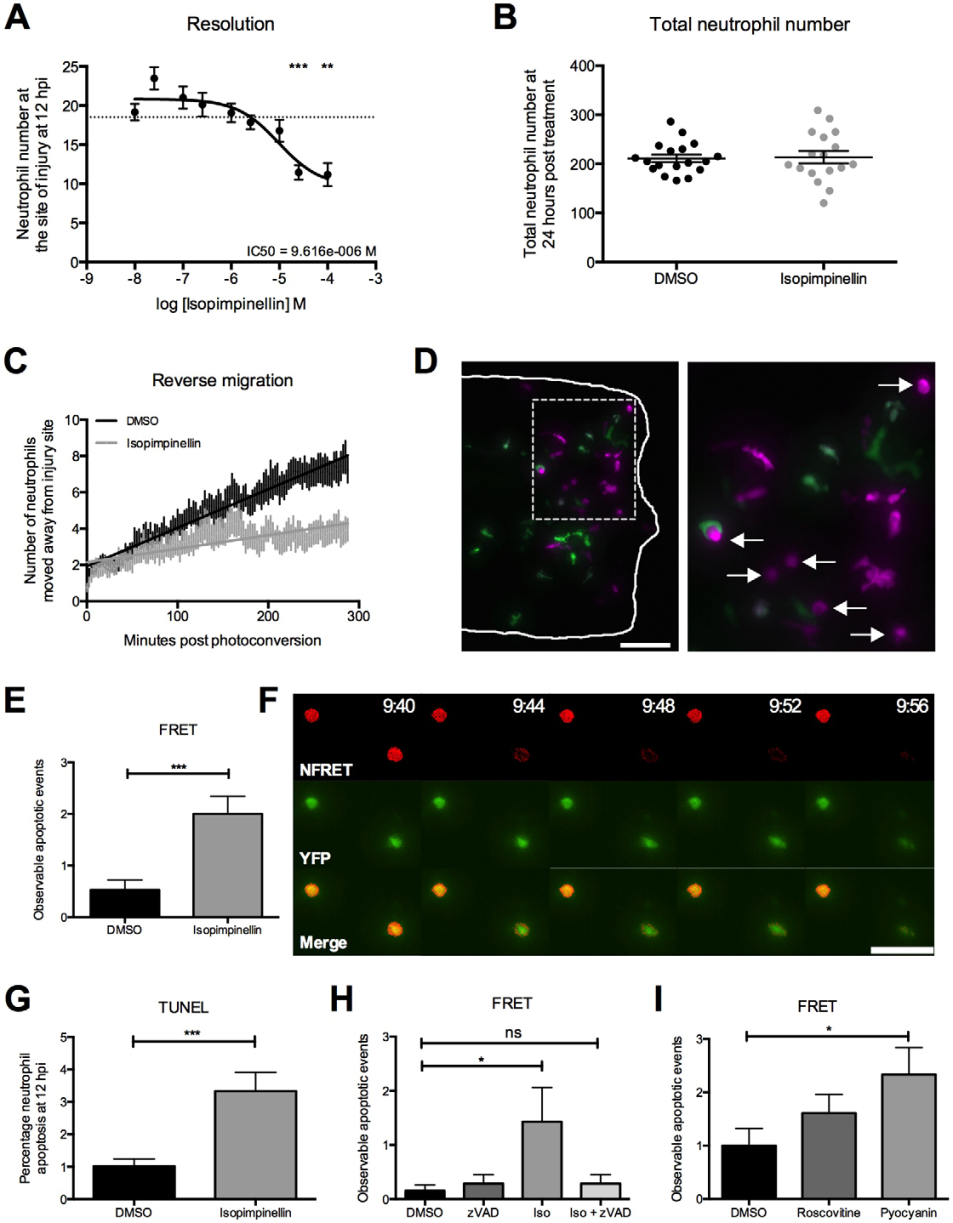
**Isopimpinellin accelerates inflammation resolution *in vivo* by inducing neutrophil apoptosis.** (A) Inflammation resolution assay in *mpx:GFP* larvae treated with varying doses of isopimpinellin at 6 hpi. Isopimpinellin significantly reduces neutrophil numbers at the wound at 12 hpi in a dose-dependent manner (one-way ANOVA with Dunnett's multiple-comparison post-test; **P<0.01, ***P<0.001; *n*=18, performed as three independent experiments). Dotted line at *y*=18.5 indicates mean neutrophil number at wound in DMSO control larvae. (B) Total neutrophil number measured in *mpx:GFP* larvae treated with DMSO or 25 μM isopimpinellin for 24 h. Isopimpinellin did not affect total neutrophil number (unpaired *t*-test; *P*=0.8696; *n*=18, performed as three independent experiments). (C) Reverse-migration assay in *mpx/Kaede* larvae treated with DMSO or 25 μM isopimpinellin from 4 hpi. Neutrophils at the site of injury were photoconverted at 6 hpi and the numbers of photoconverted cells that moved away from the wound were quantified over 5 h. Neutrophils migrated away from the wound at a slower rate in isopimpinellin-treated larvae compared to DMSO control larvae. (D) Representative image of isopimpinellin-treated *mpx/Kaede* larvae at 8 hpi (scale bar: 70 μm). Solid white line in the left panel indicates the outline of the tail-fin, and the boxed area is magnified in the right-hand panel. White arrows in magnified view indicate neutrophils that appear apoptotic. (E,F) FRET assay in *Tg(mpx:FRET)sh237* larvae treated with DMSO or 25 μM isopimpinellin from 4 hpi and imaged from 6 hpi. Cleavage of the caspase-3 target site results in separation of the fluorophores and loss of the FRET signal (red, F). Acceptor (neutrophil) fluorescence (green, F) persists for a further 10-20 min before cell death and loss of fluorophore integrity. Time is shown as hours:minutes. Scale bar: 50 μm. Number of observable apoptotic events was increased in isopimpinellin larvae (unpaired *t*-test; ***P<0.001; *n*=18, performed as three independent experiments). (G) TUNEL assay in *mpx:GFP* larvae treated with DMSO or 25 μM isopimpinellin from 6 hpi and fixed at 12 hpi. Numbers of TSA-positive neutrophils and TSA/TUNEL double-positive apoptotic neutrophils at the site of injury were measured to calculate percentage neutrophil apoptosis, which was increased in isopimpinellin-treated larvae (unpaired *t*-test; ***P<0.001; *n*=115, performed as two independent experiments). (H) Larvae were treated with DMSO, 100 μM Z-VAD-FMK (zVAD), 25 μM isopimpinellin (Iso) or in combination (Iso+zVAD) from 4 hpi and imaged from 6 hpi. Number of observable apoptotic events was increased with isopimpinellin alone but the effect was lost with the addition of Z-VAD-FMK (one-way ANOVA with Bonferroni's multiple-comparison post-test to compare selected columns; *P<0.05; ns, non-significant; *n*=14, performed as three independent experiments). (I) Larvae were treated with DMSO, 20 μM roscovitine or 50 μM pyocyanin from 4 hpi and imaged from 6 hpi. Number of observable apoptotic events was increased with pyocyanin (one-way ANOVA with Bonferroni's multiple-comparison post-test to compare selected columns; *P<0.05; *n*=18, performed as three independent experiments).

We originally identified isopimpinellin as a new pro-resolution compound in our screen for accelerators of inflammation resolution (Robertson et al., 2014). On further investigation, we found that when zebrafish larvae were exposed to isopimpinellin once inflammation was already established at 6 hpi, there was a concentration-dependent reduction in neutrophil numbers at the wound at 12 hpi (Fig. 3A). Isopimpinellin did not affect total neutrophil number in whole larvae (Fig. 3B). In our previous study, we showed that we could pharmacologically drive inflammation resolution by promoting neutrophil reverse migration (Robertson et al., 2014). To investigate whether isopimpinellin could also act via this mechanism, we photoconverted neutrophils specifically at the wound region at 6 hpi in *Tg(mpx:Gal4);Tg(UAS:Kaede)i222* larvae, as described (Elks et al., 2011; Holmes et al., 2012). However, we found that fewer photoconverted neutrophils migrated away from the wound over time in isopimpinellin-treated larvae compared to the vehicle controls (Fig. 3C).

During the course of inflammation resolution in these larvae, we often observed neutrophils adopting a rounded and condensed morphology, characteristic of the apoptotic phenotype we have previously reported (Loynes et al., 2010) (Fig. 3D). To further investigate neutrophil apoptosis as a pro-resolution mechanism in zebrafish, we developed a new *Tg(mpx:FRET)sh237* reporter line by targeting a fluorescence resonance energy transfer (FRET)-based reporter for caspase-3 activity (Tyas et al., 2000) into our myeloperoxidase bacterial artificial chromosome (BAC) (Renshaw et al., 2006). This FRET reporter consists of a CFP-YFP fluorophore pair linked by the caspase-3 cleavage sequence DEVD, such that, when caspase-3 is activated, the linker peptide is cleaved and the FRET signal is lost. Neutrophil apoptosis is caspase-3-dependent (Pongracz et al., 1999) and caspase-3 has been characterised in zebrafish, sharing 62% identity to human caspase-3 and the same substrate specificities (Yabu et al., 2001). When imaged during the resolution phase of inflammation, we detected an increase in the number of apoptotic events occurring in neutrophils at the site of injury in isopimpinellin-treated *mpx:FRET* larvae (Fig. 3E), but no apoptotic events were ever seen in the head region of the embryo, suggesting that this is an inflammation-specific response. In apoptotic cells, we observed loss of the FRET signal, indicative of caspase-3 activity, shortly after ‘cell rounding’, and the YFP acceptor fluorescence was lost 5 to 10 minutes later (Fig. 3F). The pro-apoptotic effect of isopimpinellin was validated using dual TSA/TUNEL staining, which also revealed an increase in the percentage of neutrophil apoptosis at the wound (Fig. 3G). We have previously shown that the pan-caspase inhibitor Z-VAD-FMK blocks neutrophil apoptosis, whereas pyocyanin and roscovitine can accelerate it (Loynes et al., 2010). To further illustrate the utility of this novel transgenic line, we used Z-VAD-FMK to demonstrate a reduction in the isopimpinellin-induced increase in observable apoptotic events (Fig. 3H), and pyocyanin and roscovitine to demonstrate an increase in apoptotic events (Fig. 3I), which were revealed in real time *in vivo* for the first time.

### Isopimpinellin and related disodium cromoglycate do not act as antioxidants

**Fig. 4.**
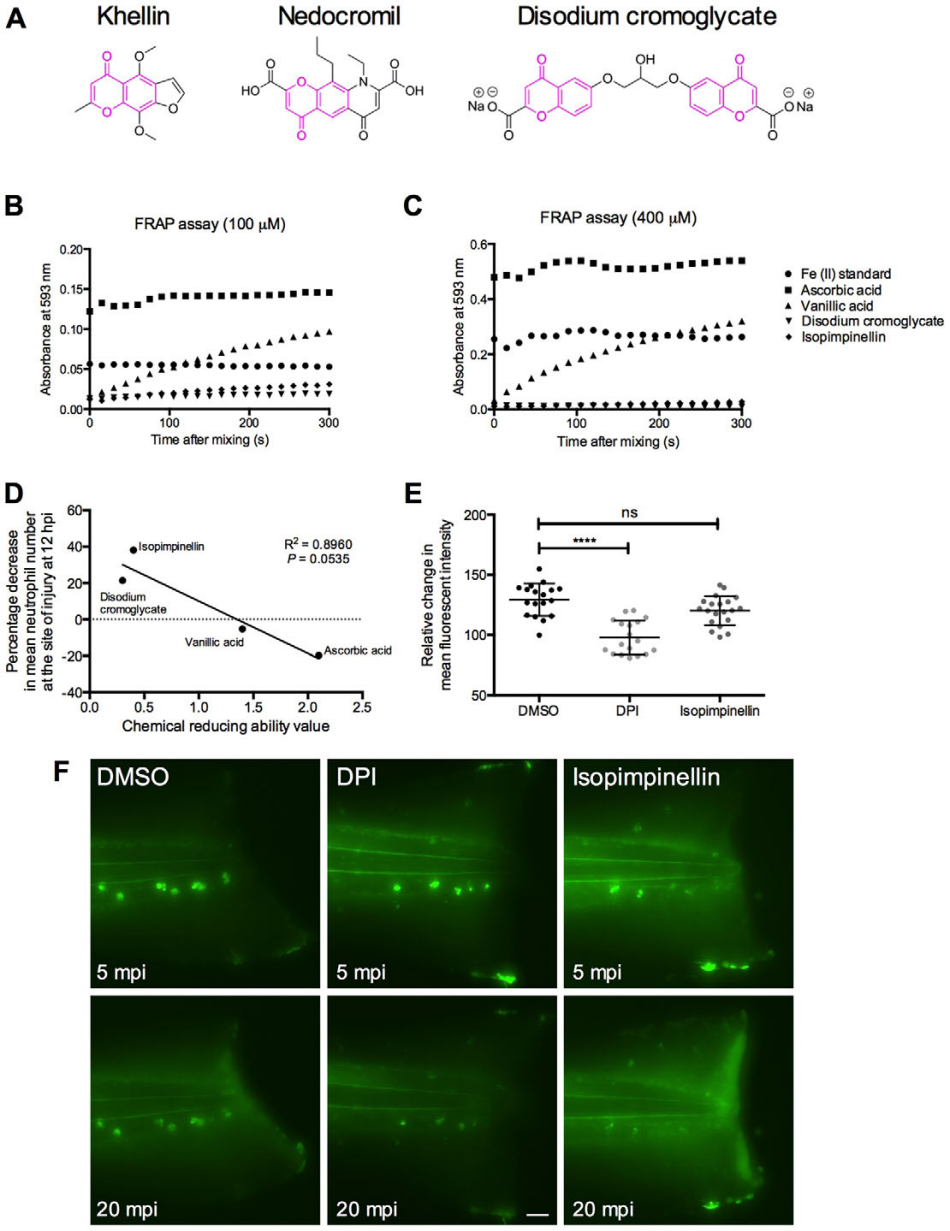
**Isopimpinellin and disodium cromoglycate do not have antioxidant activity.** (A) Isopimpinellin is a structural isomer of khellin, from which a series of functional benzopyrone analogues were designed, including disodium cromoglycate and nedocromil. (B,C) FRAP assay, performed at 37°C for 5 min. Graphs show the rate of increase in absorbance at 593 nm, corrected for reagent blank readings, at (B) 100 μM and (C) 400 μM of each compound, as indicated. (D) The *in vivo* activity of the compounds tested (expressed as the percentage decrease in the mean neutrophil numbers at the wound at 12 hpi), in comparison to the vehicle control (refers to data from Figs 3A, 6A and Fig. S3), plotted against the chemical reducing ability value (refers to data from Table S1) (linear regression analysis; *R*^2^=0.8960; *P*=0.535). (E) *In vivo* antioxidation assay. Larvae were pretreated with DMSO, isopimpinellin or DPI for 2 h and pentafluorobenzenesulfonyl fluorescein for 1 h prior to tail-fin injury. Imaging commenced 5 min post-injury (mpi) and mean fluorescence intensity at the injury site was measured at 5 and 20 mpi. Mean fluorescent intensity at 20 mpi is expressed as a percentage of the mean fluorescent intensity at 5 mpi. This was significantly reduced by DPI but isopimpinellin had no effect (one-way ANOVA with Dunnett's multiple-comparison post-test; ****P<0.0001; ns, non-significant; *n*>15, performed as two independent experiments). Illustrative images are shown in F. Scale bar: 50 μm.

Isopimpinellin is structurally related to khellin, a naturally occurring benzopyrone also found in plants of the Apiaceae family. Originally used in ancient Egyptian folk medicine, it was discovered in the mid-1900s that khellin in its isolated form was an effective treatment for bronchial asthma, and this led to the development of a series of functional benzopyrone analogues, including disodium cromoglycate and nedocromil (Fig. 4A), collectively termed the ‘cromones’, which are now in clinical use (Edwards and Howell, 2000). These and other structurally related coumarins and flavonoids have been reported to possess antioxidant activity (Bubols et al., 2013). To explore this as a potential mechanism of action for our compounds, we analysed their reducing ability using the ferric reducing ability of plasma (FRAP) assay (Benzie and Strain, 1996). Antioxidants are preferentially oxidised over another substrate, and their oxidation results in the reduction of another component. Therefore, a good antioxidant will also be a good reducing agent. Using this method, we found that the known antioxidant ascorbic acid showed strong reducing ability, at levels consistent with previous studies (Benzie and Strain, 1996), and vanillic acid also exhibited substantial reducing ability. However, neither isopimpinellin nor disodium cromoglycate showed appreciable reducing ability in this assay, suggesting that these compounds are unlikely to be acting as antioxidants in our studies (Fig. 4B,C and Table S1). Furthermore, when we compared the relative chemical reducing ability of these four compounds with their effect on the resolution of inflammation *in vivo*, we found that there was actually an inverse correlation between biological activity and chemical reducing ability (Fig. 4D). Our most active pro-resolution compounds *in vivo* showed little chemical reducing ability, whereas neither ascorbic acid nor vanillic acid, known antioxidants, exhibited a pro-resolution effect in our model (Fig. S3). To explore this in an *in vivo* setting, we measured the effect of isopimpinellin on hydrogen peroxide (H_2_O_2_), an important reactive oxygen species (ROS) signal known to promote neutrophil accumulation following tissue injury (Niethammer et al., 2009). Using a ROS sensor (Rieger and Sagasti, 2011), there was no reduction in the intensity of the H_2_O_2_ gradient at the tail-fin wound in zebrafish larvae pretreated with isopimpinellin, in contrast to the potent effect observed in the presence of the NAPDH oxidase inhibitor diphenyleneiodonium (DPI) (Fig. 4E,F). Taken together, these data suggest that this subset of compounds do not exhibit their activity by acting as antioxidants in our zebrafish inflammation assays.

### Isopimpinellin and related cromones do not induce human neutrophil apoptosis *in vitro*

**Fig. 5.**
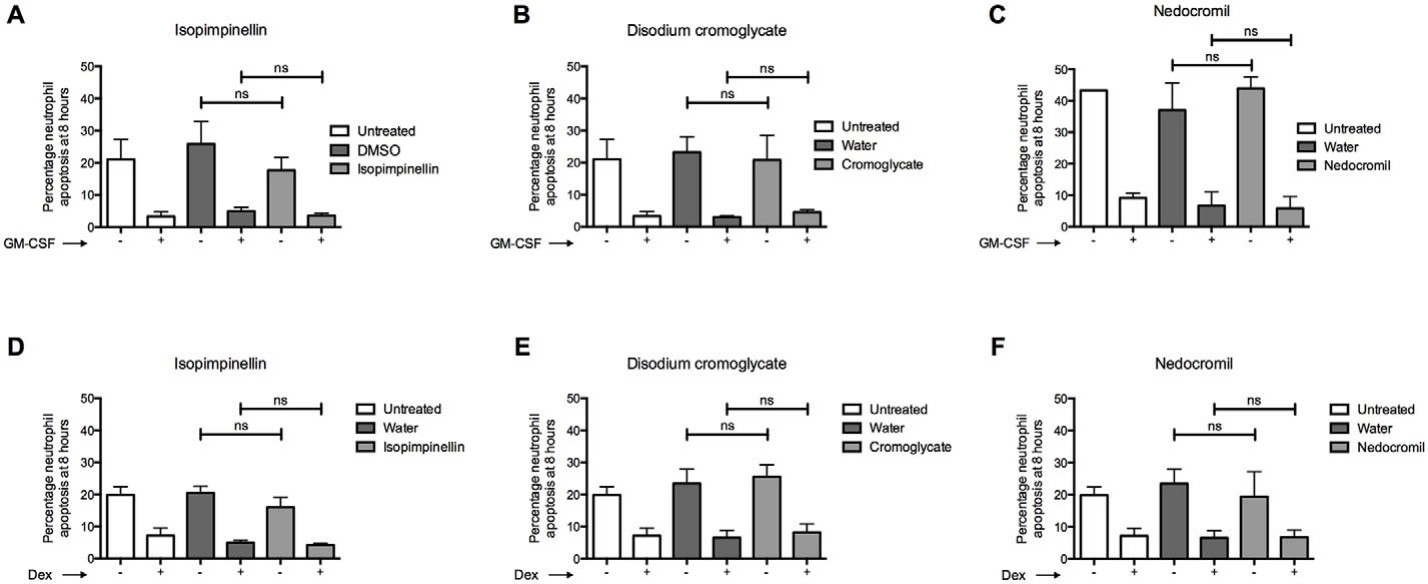
**Isopimpinellin and related clinical cromones have no effect on human neutrophil apoptosis *in vitro*.** (A-F) Human neutrophils were isolated from whole blood and incubated for 8 h with (+) or without (−) 0.01 μg/ml GM-CSF or 1 μM dexamethasone (Dex) as indicated, and 10 μM (A,D) isopimpinellin, (B,E) disodium cromoglycate or (C,F) nedocromil. In each case, two cytospins (technical replicates) were acquired per condition and the percentage of neutrophil apoptosis was calculated based on nuclear morphology. Experiments in A,B and D-F were performed at the same time using the same controls. Isopimpinellin, disodium cromoglycate and nedocromil did not induce neutrophil apoptosis compared to their vehicle controls, or inhibit neutrophil survival induced by either GM-CSF or dexamethasone [one-way ANOVA with Bonferroni's multiple comparison post-test to compare selected columns; *P*>0.05 for all comparisons (ns, non-significant); *n*=2, performed as independent experiments from two different donors performed on different days].

The precise anti-inflammatory mechanisms of the cromones are not fully defined but are likely to be mediated by secretion of the calcium and phospholipid-binding protein, Annexin A1 (AnxA1) (Yazid et al., 2009, 2010). AnxA1 is thought to induce neutrophil apoptosis in a caspase-3-dependent manner and might also function as an ‘eat me’ signal to promote the phagocytosis of apoptotic neutrophils by macrophages (Arur et al., 2003; Scannell et al., 2007; Vago et al., 2012). Having observed a pro-apoptotic effect on zebrafish neutrophils *in vivo*, we hypothesised that isopimpinellin and the related clinical cromones might act similarly on human neutrophils, and that this might be a previously unidentified pro-resolution mechanism of this series of compounds. Neutrophils were freshly isolated from whole blood and incubated with isopimpinellin, disodium cromoglycate or nedocromil in either the presence or absence of the neutrophil survival signal granulocyte-macrophage colony-stimulating factor (GM-CSF). Unexpectedly, after 8 hours, we found no difference in the percentage of apoptosis in neutrophils exposed to any of the three compounds compared to their vehicle control and none of them were able to override the survival effect of GM-CSF (Fig. 5A-C). It is known that neutrophil lifespan is prolonged by glucocorticoids (Heasman et al., 2003; Liles et al., 1995) and it has been suggested that AnxA1 acts as a downstream modulator of their effects during the resolution phase of inflammation, by enhancing neutrophil apoptosis and efferocytosis (Vago et al., 2012; Dalli et al., 2013). We therefore examined the effects of isopimpinellin, disodium cromoglycate and nedocromil on neutrophil apoptosis in the presence of dexamethasone. However, we did not detect increased apoptosis in neutrophils exposed to dexamethasone in combination with any of our compounds (Fig. 5D-F). Although these experiments were not powered to detect a small effect, we have excluded a difference of comparable magnitude to the *in vivo* data.

### Cromones in clinical use might act indirectly to induce neutrophil apoptosis *in vivo*

**Fig. 6.**
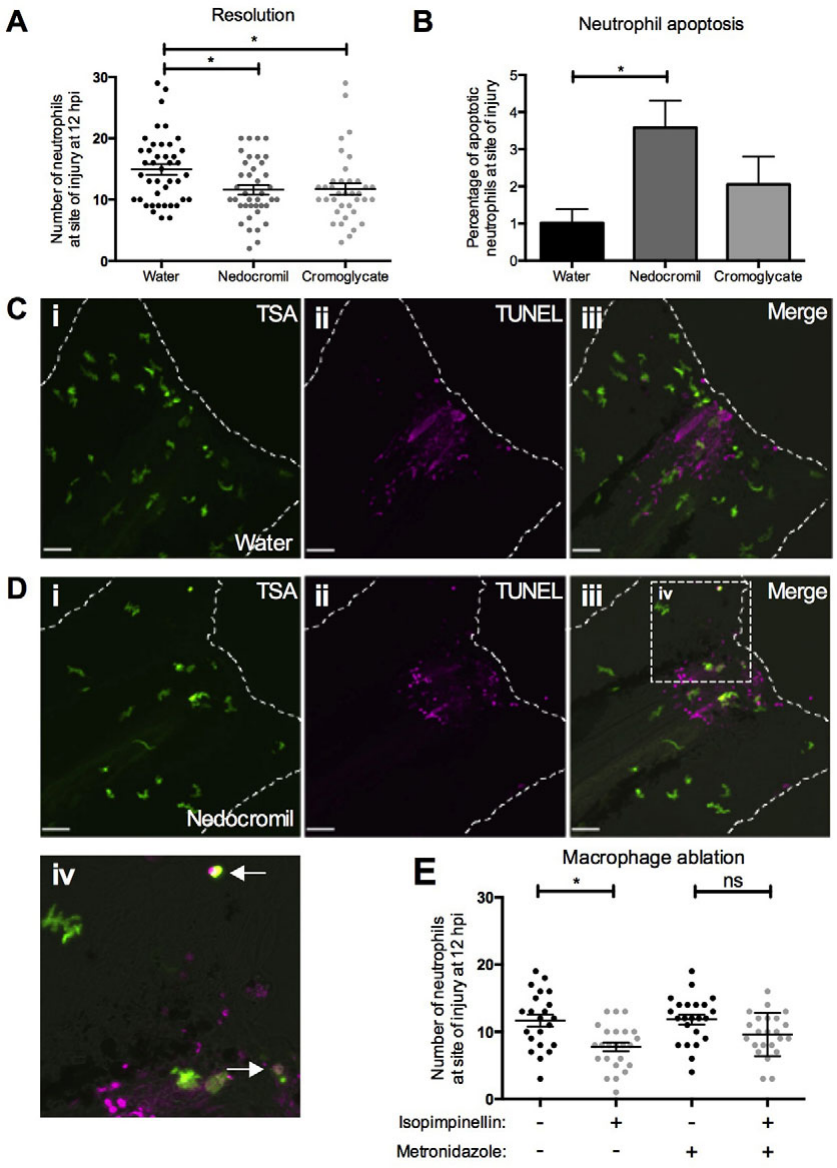
**Clinical cromones promote neutrophil apoptosis to drive inflammation resolution *in vivo*.** (A) Inflammation-resolution assay in *mpx:GFP* larvae injected with 0.4 pg/μl nedocromil or 0.5 pg/μl disodium cromoglycate at 6 hpi. Both compounds significantly reduce neutrophil numbers at the wound at 12 hpi compared to the water control (one-way ANOVA with Dunnett's multiple-comparison post-test; *P<0.5; *n*>36, performed as four independent experiments). (B-D) TUNEL assay in *mpx:GFP* larvae injected with water, 0.4 pg/μl nedocromil or 0.5 pg/μl disodium cromoglycate from 6 hpi and fixed at 12 hpi. Numbers of TSA-positive neutrophils and TSA/TUNEL double-positive apoptotic neutrophils at the site of injury were measured to calculate percentage neutrophil apoptosis, which was increased in nedocromil-treated larvae (one-way ANOVA with Dunnett's multiple-comparison post-test; *P<0.05; *n*>54, performed as three independent experiments). (C,D) Illustrative images of water-injected (C) and nedocromil-injected (D) larvae following TSA/TUNEL staining (scale bars: 40 μm). Broken lines indicate the outline of the tail-fin. White arrows in magnified view of boxed area in Diii (Div) indicate apoptotic neutrophils, identified by morphology and double TSA/TUNEL labelling. (E) Inflammation-resolution assay in the absence of macrophages. Metronidazole ablation of macrophages impairs the effect of isopimpinellin (one-way ANOVA with Bonferroni's multiple-comparison post-test to compare selected columns; *P<0.05; ns, non-significant; *n*>20; performed as three independent experiments).

Our data suggest that isopimpinellin and the related clinical cromones do not have a direct effect on neutrophil survival. We suspected that this might be explained by the lack of a required stimulus in our *in vitro* culture system because we had already observed an increase in neutrophil apoptosis during inflammation resolution in zebrafish larvae exposed to isopimpinellin (Fig. 3). To similarly investigate the effects of disodium cromoglycate and nedocromil *in vivo*, we performed inflammation resolution assays and TSA/TUNEL staining in *mpx:GFP* larvae. Because these cromones are inactive when given by immersion (data not shown) (Yazid et al., 2010), we administered them at 6 hpi by vascular injection into the Duct of Cuvier, a technique that has been used previously for bacterial infection (Benard et al., 2012). At 12 hpi, neutrophil numbers at the wound were reduced following injection with either nedocromil or disodium cromoglycate, in comparison to the vehicle control (Fig. 6A). We also found a significant increase in the percentage of apoptosis of neutrophils at the wound in nedocromil-injected larvae (Fig. 6B-D). The lack of effect in isolated human neutrophils *in vitro* might be explained by an indirect effect of the compound acting on neutrophils via another cell type, such as macrophages. To test this, we used a combined genetic/pharmacological macrophage ablation system, in which transgenic expression of bacterial nitroreductase is driven by the *mpeg* promoter and larvae are exposed to metronidazole treatment (Prajsnar et al., 2012). When macrophages were partially ablated in this manner, the effect of isopimpinellin was less significant, indicating a partial dependence on macrophages for the isopimpinellin response (Fig. 6E). Taken together, these data suggest that isopimpinellin and the clinically available cromones share both structural and functional similarity, acting to accelerate inflammation resolution *in vivo* by indirect induction of neutrophil apoptosis.

## DISCUSSION

Neutrophil-dominated inflammatory diseases remain a large, worldwide problem and there is a major unmet need for more effective treatments. Drug discovery strategies focus on targeting the mechanisms that regulate neutrophil recruitment and clearance during inflammation resolution, and recent efforts to identify new anti-inflammatory therapeutics have made use of the zebrafish model (d'Alencon et al., 2010; Robertson et al., 2014). Many features of the innate immune system are highly conserved between zebrafish and mammalian species (Trede et al., 2004). In our tail-fin injury assay, the cellular components and kinetics of the acute inflammatory response are comparable to those in mammalian systems, making this a useful model to study inflammation and dissect the mechanisms that might become disrupted to cause inflammatory disease (Renshaw et al., 2006).

In this study, we investigated the mechanism of action of a group of structurally similar compounds that we identified in our recent *in vivo* screen for accelerators of inflammation resolution (Robertson et al., 2014). We describe the anti-inflammatory and pro-resolution activity of isopimpinellin, which, in our assays, was the most active compound within this subset. Based on our previous hierarchical cluster analysis, we were able to accurately predict that the mechanism by which isopimpinellin inhibits neutrophil recruitment is dependent on PI3K signalling. This explains, at least in part, the differences in chemotactic behaviour that we observed during the recruitment phase of the inflammatory response. Over the course of our time-lapses, neutrophils from isopimpinellin-treated larvae did not migrate preferentially towards the wound and did not travel as quickly or as far as neutrophils from control larvae. Directed neutrophil migration relies on polarisation of the cell, which is dependent on localised PI3K signalling at the leading edge. Using the transgenic *lyz/PHAkt* reporter line, we showed that isopimpinellin treatment resulted in a loss of the plasma-membrane localisation of PHAkt-EGFP and a rounded-tail neutrophil phenotype, similar to the effect of the known PI3K inhibitor LY294002 that we and others have previously observed (Yoo et al., 2010; Wang et al., 2014; Burgon et al., 2014). Interestingly, LY294002 is structurally similar to the compounds we have investigated in this study, and was originally developed as an analogue of quercetin, a benzopyrone that inhibits PI3K by blocking the ATP-binding site (Vlahos et al., 1994). We measured a reduction in the polarity index of neutrophils from both isopimpinellin-treated larvae and those treated with another compound from our structurally similar subset, xanthone. Our data suggest that these compounds act either directly on or upstream of PI3K, resulting in reduced phosphorylation and translocation of the PH domain of Akt to the plasma membrane, and disruption of directional migration towards the wound.

Alongside its inhibitory effect on neutrophil recruitment, we found that isopimpinellin also accelerated inflammation resolution *in vivo*. Our data suggest that this occurs as a result of increased neutrophil apoptosis, rather than reverse migration, which is perhaps to be expected given that PI3K is also thought to be important for neutrophil migration away from a wound (Yoo et al., 2010). Neutrophil apoptosis followed by phagocytic uptake by macrophages is the best-described process by which neutrophils are removed during inflammation resolution (Savill et al., 1989). Current approaches to reliably detect neutrophil apoptosis as it occurs *in vivo* are limited. Although it is possible to label apoptotic neutrophils in zebrafish larvae post-fixation, this provides only a snapshot of the entire resolution phase. Here, we have developed a new *in vivo* FRET reporter line for caspase-3 activity that enables us to visualise neutrophil apoptosis in real time. When caspase-3 is activated in neutrophils, the FRET signal is lost, prior to and independently of the loss of GFP we have seen previously when neutrophils undergo apoptosis in our *mpx:GFP* zebrafish (Loynes et al., 2010). Our data indicate that the pro-apoptotic effect of isopimpinellin is caspase-3-dependent and, although neutrophil apoptosis occurs rarely in zebrafish larvae during the inflammatory response, it is possible to pharmacologically manipulate this process to promote resolution. Our new reporter line could provide a useful tool for studying the mechanisms regulating neutrophil apoptosis *in vivo* and for the identification of compounds that induce neutrophil apoptosis.

Intriguingly, the clinically available mast cell stabilisers disodium cromoglycate and nedocromil are benzopyrone derivatives with incompletely defined anti-inflammatory activity. Given their structural similarity to isopimpinellin, we hypothesised that neutrophil apoptosis might be a previously unidentified mechanism of action for these compounds. In our human neutrophil assays, we did not detect any change in the rate of apoptosis in the presence of isopimpinellin or either of the clinical cromones. This was unexpected given that similar compounds have been reported to induce neutrophil apoptosis *in vitro* and to override survival factors including GM-CSF and dexamethasone (Lucas et al., 2013). It is possible that subtle differences in the chemical structures of the benzopyrone derivatives have profound effects on their biological activity, for example by affecting target binding, metabolism or their ability to permeate the cell membrane. Because we were unable to detect an increase in neutrophil apoptosis with nedocromil or disodium cromoglycate *in vitro*, we cannot speculate on the involvement of AnxA1, the release of which can be induced by these compounds (Yazid et al., 2010). Given our evidence that nedocromil and isopimpinellin can induce neutrophil apoptosis and promote inflammation resolution *in vivo*, we suspect that another stimulus or cell type might be important for mediating their activity. Our macrophage ablation experiments suggest that the effect of isopimpinellin might be at least partially dependent on macrophages. Further investigation is required to dissect this mechanism and establish the effect of nedocromil in the absence of these cells. Disodium cromoglycate also accelerated inflammation resolution in our system, although we were unable to confirm that this was due to an increase in neutrophil apoptosis. Nedocromil was developed as a more potent and stable alternative to disodium cromoglycate (Edwards and Stevens, 1993), which we suspect could explain the difference in activity that we observed between these two compounds.

The mechanism of action of isopimpinellin and related chromones and coumarins remains to be determined. Our FRAP studies indicate that the active compounds we tested do not act as antioxidants through single-electron transfer. Although these results alone do not rule out the possibility that they might act as oxygen-radical scavengers, when taken together with our *in vivo* data, an antioxidant mechanism seems unlikely. We did not observe a reduction in the H_2_O_2_ gradient at the site of injury in isopimpinellin-treated larvae, in comparison to those treated with the NADPH oxidase inhibitor, DPI. However, the ROS sensor used in these experiments is not specific to H_2_O_2_ and we cannot rule out the possibility that isopimpinellin might reduce the levels of other ROS. A recent report describes antagonism of the AnxA1 receptor, formyl peptide receptor 1 (FPR1), by a series of benzopyrone analogues (Schepetkin et al., 2014). The precise functions of AnxA1 and FPR1 signalling during inflammation resolution have not yet been well-established and we suspect that the effects of FPR1 activation over the course of the inflammatory response might be temporally dependent. An alternative mechanism of action for the cromones has also been proposed. Two research groups have demonstrated that disodium cromoglycate and nedocromil might activate the G-protein-coupled receptor GPR35 to induce calcium mobilisation, inositol phosphate accumulation and β-arrestin-2 recruitment in transfected cells (Jenkins et al., 2010; Yang et al., 2010).

In summary, we have identified a series of lead compounds with the potential both to limit the further recruitment of neutrophils to areas of inflammation and also to promote the clearance of persistent neutrophils that are already *in situ*. This combined anti-inflammatory and pro-resolution activity might make these compounds particularly valuable for the treatment of chronic inflammatory diseases. Further investigation is necessary to determine the precise mechanism of action of isopimpinellin and other benzopyrone analogues *in vivo*.

## MATERIALS AND METHODS

### Reagents

Isopimpinellin (MicroSource Discovery Systems Inc., Gaylordsville, CT, USA) was used at 25 μM unless indicated otherwise. Xanthone was used at 25 μM unless stated otherwise, LY294002 at 50 μM, pyocyanin at 50 μM and disodium cromoglycate at 0.5 pg/μl (all obtained from Sigma-Aldrich, Poole, UK). Roscovitine was used at 20 μM (New England Biolabs, Hitchin, UK) and Z-VAD-FMK at 100 μM (Calbiochem, Manchester, UK). Nedocromil was used at 0.4 pg/μl and was a kind gift from Professor Rod Flower (William Harvey Research Institute, London, UK). The related benzopyrones anthraquinone, 4-chromanone, 1,2,3,4-tetrahydranaphthalene and xanthene were also obtained from Sigma-Aldrich, whereas anthrone and dihydrocoumarin were acquired from Alfa Aesar (Heysham, UK) and α-tetralone from L. Light & Co. Ltd (Colnbrook, UK). GM-CSF (from PeproTech, London, UK) was used at 0.01 μg/ml. Zebrafish were treated with compounds by immersion, with the exceptions of disodium cromoglycate and nedocromil, which were administered by vascular injection into the Duct of Cuvier, alongside fluorescein as a marker for successful injection. DMSO (Sigma-Aldrich) or water were used as vehicle controls, as indicated.

### Analogue compound synthesis

Compounds were synthesised according to literature procedures as follows: MMM101 using a slightly modified procedure (Pirkle and Finn, 1983); MMM103 (Niwa et al., 2008); MMM115 (Fougerousse et al., 2000; Hirao et al., 1984); MMM116P using an adapted method (Mouysset et al., 1988); MMM117 using a modified procedure (Bird et al., 1983).

### Transgenic zebrafish generation and maintenance

Zebrafish were raised and maintained according to standard protocols (Nusslein-Volhard and Dahm, 2002), in UK Home Office-approved aquaria at the Bateson Centre, University of Sheffield. The neutrophil-specific zebrafish line *Tg(mpx:GFP)i114* (Renshaw et al., 2006), referred to as *mpx:GFP*, was used for all experiments unless stated otherwise. All procedures were performed on larvae at 3 days post-fertilisation (dpf). Inflammation was initiated by tail-fin transection and neutrophil numbers at the wound were recorded following our standard protocols (Renshaw et al., 2006; Robertson et al., 2014). The *Tg(mpx:FRET)sh237* line, referred to as *mpx:FRET*, was generated by BAC recombineering as previously described (Renshaw et al., 2006), using a caspase-3-specific FRET reporter (Tyas et al., 2000).

### *In vivo* neutrophil recruitment assays

For neutrophil recruitment assays, *mpx:GFP* larvae were treated with compounds at the dose indicated immediately after wounding and numbers of neutrophils at the site of injury were counted at 6 h post-injury (hpi). To track neutrophil migration during the recruitment phase of inflammation, larvae were pretreated for 2 h prior to tail-fin transection, followed by mounting in 0.8% low-melting-point agarose containing the appropriate drug at 1 hpi. A 2-h time-lapse series was captured and neutrophils were manually tracked using Volocity™ imaging software (PerkinElmer Life and Analytical Sciences, Cambridge, UK) for analysis of speed, meandering index, displacement and bearing, as previously described (Elks et al., 2011; Robertson et al., 2014). PI3K assays were performed using *Tg(lyz.PHAkt:GFP)i277* larvae, which were pre-incubated with test compounds for 2 h, followed by wounding, imaging and polarity analysis as described (Wang et al., 2014).

### *In vivo* inflammation-resolution assays

For inflammation-resolution assays, *mpx:GFP* larvae were treated at 6 hpi once inflammation was already established, and neutrophil numbers at the wound were counted at 12 hpi. Total neutrophil numbers were analysed in uninjured larvae following compound treatment for 24 h, as previously described (Robertson et al., 2014). Images were converted to 8-bit, binary TIFs using ImageJ (NIH), and the ‘Measure’ function was used to count the number of neutrophils in an automated manner. Reverse migration assays were performed using *Tg(mpx:Gal4);Tg(UAS:Kaede)i222* larvae following established methods (Elks et al., 2011; Holmes et al., 2012). Neutrophil apoptosis was measured in paraformaldehyde-fixed larvae following Tyramide Signal Amplification staining (TSA™-Plus, PerkinElmer) to label neutrophil myeloperoxidase and using an ApopTag^®^ Red *In Situ* Apoptosis Detection Kit (TUNEL) (Millipore Corporation, Herts, UK) to label apoptotic cells, as previously described (Elks et al., 2011). The percentage of neutrophil apoptosis was measured using Volocity™ software.

### FRET assay

Tail-fin transection was performed on *Tg(mpx:FRET)sh237* larvae at 3 dpf followed by treatment with compounds at 4 hpi. Larvae were mounted and FRET imaging was performed from 6 hpi. Embryos were imaged for 6 h on a TE-2000U microscope (Nikon, Japan) with an Orca-AG Camera (Hamamatsu, Japan) using Volocity™ imaging software. Donor fluorescence was imaged with a D436/20× CFP excitation filter and a D480/40m CFP emission filter. Acceptor fluorescence was imaged with a HQ500/20× YFP excitation filter and an HQ535/30m emission filter. FRET images were taken with the CFP excitation and the YFP emission filters, using a 455DCLP dichroic mirror (Chroma, Germany). Spectral bleed-through constants were calculated using fixed HEK293T cells transfected with either p1CFP or p1YFP and mounted in VECTASHIELD mounting media (Vector Laboratories, Peterborough, UK). Volocity™ was used to calculate NFRET (normalised FRET value) (Xia and Liu, 2001).

### *In vivo* antioxidation assays

At 3 dpf, zebrafish larvae (*nacre*) were treated with DMSO, isopimpinellin or DPI (100 μM, Sigma-Aldrich) for 2 h prior to injury. Incubation in the appropriate compound was continued during injury and subsequent imaging. During the pretreatment period, the larvae were also incubated in pentafluorobenzenesulfonyl fluorescein (10 μM, Santa Cruz Biotechnology, Santa Cruz, CA, USA) in the dark for 1 h. The dye was removed prior to mounting. After 2 h pretreatment, larvae were mounted in agarose containing the appropriate compound. A window was cut in the agarose to allow prompt imaging of the larval tail after injury. Tail-fin transection was performed and imaging commenced at 5 min post-injury (mpi). Mean fluorescent intensity at the injury site was measured at 5 mpi and 20 mpi using ImageJ. Mean fluorescent intensity at 20 mpi was expressed as a percentage of the mean fluorescent intensity at 5 mpi.

### Ferric reducing ability of plasma (FRAP) assays

The method of Benzie and Strain (1996) was followed with slight modifications. 300 mM acetate buffer (pH 3.6) was prepared from 3.1 g sodium acetate trihydrate (Alfa Aesar, Heysham, UK) and 16 ml glacial acetic acid (VWR International, Lutterworth, UK) made up to 1 litre with distilled water. Other reagents used were 10 mM 2,4,6-Tris(2-pyridyl)-s-triazine (TPTZ) (Sigma-Aldrich) in 40 mM hydrochloric acid (VWR International), and aqueous 20 mM iron (III) chloride hexahydrate (BDH Laboratory Supplies, Poole, UK). FRAP reagent was prepared freshly as required, by mixing 10 ml acetate buffer, 1 ml TPTZ solution and 1 ml iron (III) chloride hexahydrate solution. Aqueous standard solutions of iron (II) at concentrations of 100, 200, 400, 600, 800 and 1000 µM were prepared using iron (II) sulfate heptahydrate (Sigma-Aldrich), and their absorbances at 593 nm at 37°C were used for calibration of the assay, using a line of best fit as determined by linear regression analysis. Solid L-(+)-ascorbic acid, vanillic acid and disodium cromoglycate (all obtained from Sigma-Aldrich) were tested as aqueous solutions, whereas isopimpinellin was used as an ethanolic solution. All compounds were tested at both 100 µM and 400 µM. All UV-visible spectrometry was carried out using a Cary 50 Probe UV-Visible Spectrophotometer (Varian) pre-warmed to 37°C using a water bath, and in a quartz cuvette of 1-cm path length. Absorbance values at 593 nm were plotted using the Cary WinUV Kinetics application (Varian, version 3.00). 3 ml freshly prepared FRAP reagent in a quartz cuvette was warmed to 37°C, and a blank reading was taken at 593 nm. A 100 µl sample of compound solution was then added, and absorbance readings were taken after 0.1 s and then at 15-s intervals for a total of 5 min (after which the absorbance values for most samples had stabilised). These were then corrected relative to the blank reading. The absorbance value of the sample at 5 min was then used for all future calculations. For each sample, the effective iron (II) concentration in solution corresponding to that absorbance value was determined, using the calibration graph (described above). This value was then compared to the corresponding iron (II) standard value for the same concentration of reagent (i.e. 100 or 400 µM), and was expressed as a ratio, for each of the two concentrations tested. These ratios were then used to determine the mean ratio for each compound, referred to as the chemical reducing ability value.

### Macrophage ablation experiments

Zebrafish larvae from *Tg(mpeg:gal4)SH256; Tg(UAS:nfsB.mCherry)C264; Tg(mpx:GFP)i114* zebrafish were raised to 2 dpf and then incubated with or without metronidazole (5 mM) for approximately 16 h. At 3 dpf, tail-fin transection was performed and larvae were returned to metronidazole (1.25 mM) or vehicle only for the remainder of the experiment. At 6 hpi, larvae with a good inflammatory response were treated with isopimpinellin or DMSO. At 12 hpi, the number of neutrophils at the site of injury was counted. All groups were kept in the dark throughout the experiment.

### Human neutrophil apoptosis assay

Peripheral blood neutrophils were purified using the Percoll method of separation, as described previously (Haslett et al., 1985), in accordance with the South Sheffield Research Ethics Committee (reference number: STH13927). Rates of neutrophil apoptosis based on morphology were counted on cytospins stained with Quick-Diff (Gentaur, Brussels, Belgium).

### Statistical analysis

Data were analysed (Prism 6.0; GraphPad Software, CA, USA) using unpaired, two-tailed *t*-tests for comparisons between two groups and one-way ANOVA (with appropriate post-test adjustment) for other data. In all cases, mean±s.e.m. are shown and procedures were performed and analysed blind to experimental conditions.
